# Beyond PACIFIC: Real-World Outcomes of Adjuvant Durvalumab According to Treatment Received and PD-L1 Expression

**DOI:** 10.3390/curroncol30080543

**Published:** 2023-08-08

**Authors:** Marie-Hélène Denault, Jamie Feng, Shelley Kuang, Aria Shokoohi, Bonnie Leung, Mitchell Liu, Eric Berthelet, Janessa Laskin, Sophie Sun, Tina Zhang, Cheryl Ho, Barbara Melosky

**Affiliations:** 1 BC Cancer, Vancouver Centre, 600 West 10th Avenue, Vancouver, BC V5Z 4E6, Canada; 2 Centre de Recherche de l’Institut Universitaire de Cardiologie et de Pneumologie de Québec, 2725 Ch Ste-Foy, Québec, QC G1V 4G5, Canada

**Keywords:** locally advanced non-small cell lung cancer, durvalumab, adjuvant, immune checkpoint inhibitors, PD-L1

## Abstract

Adjuvant durvalumab after chemoradiotherapy (CRT) is the standard of care for unresectable stage III non-small cell lung cancer (NSCLC). A post hoc exploratory analysis of PACIFIC revealed no OS benefit in the PD-L1 < 1% subgroup. This retrospective analysis assesses the real-world impact of durvalumab on OS according to PD-L1 tumor proportion score (TPS). Patients with stage III, unresectable NSCLC treated by CRT, with available PD-L1 TPS, from 1 March 2018 to 31 December 2020, at BC Cancer, British Columbia, Canada were included. Patients were divided into two groups, CRT + durvalumab and CRT alone. OS and PFS were analyzed in the PD-L1 ≥ 1% and <1% subgroups. A total of 134 patients were included in the CRT + durvalumab group and 117, in the CRT alone group. Median OS was 35.9 months in the CRT + durvalumab group and 27.4 months in the CRT alone group [HR 0.59 (95% CI 0.42–0.83), *p* = 0.003]. Durvalumab improved OS in the PD-L1 ≥ 1% [HR 0.53 (95% CI 0.34–0.81), *p* = 0.003, *n* = 175], but not in the <1% subgroup [HR 0.79 (95% CI 0.44–1.42), *p* = 0.4, *n* = 76]. This retrospective study demonstrates a statistically significant improvement in OS associated with durvalumab after CRT in PD-L1 ≥ 1%, but not PD-L1 < 1% NSCLC. Variables not accounted for may have biased the survival analysis. A prospective study would bring more insight.

## 1. Introduction

Lung cancer is the leading cause of cancer-related death worldwide, with a five-year survival of only 22% in Canada [1]. Prognosis depends on clinical and pathological characteristics, including stage at diagnosis which influences treatment modality and potential for cure [2,3]. About two-thirds of new cases of lung cancer are unfortunately found at an advanced stage [1,4,5]. Stage III non-small cell lung cancer (NSCLC) is a heterogeneous group of patients with either large tumors, satellite nodules in ipsilateral lobe(s), significant structural invasion and/or mediastinal node involvement [6]. Patients can potentially benefit from curative treatment by either surgery or chemoradiotherapy (CRT), depending on the presentation. For patients treated with CRT, the PACIFIC trial has been a game changer over the last few years, demonstrating that a year of adjuvant durvalumab following CRT was associated with a 5-year overall survival (OS) rate of 42.9%, an absolute 10% increase compared to placebo [7,8]. The PACIFIC regimen has since become the standard of care.

The initial results showed benefits across all levels of PD-L1 expression, defined as tumor proportion score (TPS) of ≥25%, <25% and unknown. An unplanned, posthoc analysis based on PD-L1 TPS ≥ 1% and <1% cutoffs raised questions about patient selection [9]. Compared to placebo, durvalumab improved OS in the PD-L1 ≥ 1% subgroup, but not in the PD-L1 < 1% subgroup [9]. Randomization was not stratified for PD-L1 TPS, however, which was unknown in 36.7% of patients. As a result, the durvalumab arm of the PD-L1 < 1% subgroup was disadvantaged with respect to other prognostic variables. Moreover, the trial was not powered for this subgroup analysis. Controversy and practice differences around adjuvant durvalumab in PD-L1 negative patients arose nonetheless. The European Medicines Agency approved adjuvant durvalumab following CRT in stage III NSCLC for patients with PD-L1 TPS ≥ 1% [10], while the Food and Drug Administration (FDA) and Health Canada granted approval regardless of PD-L1 expression [11,12].

This study aimed to investigate real-world outcomes of adjuvant durvalumab after CRT in stage III NSCLC according to PD-L1 expression in a publicly funded healthcare system.

## 2. Materials and Methods

BC Cancer is a provincial cancer care program serving a population of 5.1 million in British Columbia, Canada. BC Cancer has completed records on the prescribing of all cancer therapies in the province. A retrospective chart review of all stage III NSCLC patients treated with curative-intent CRT between 1 March 2018 and 31 December 2020, was conducted. Patients who had stage II or IV of the disease, a dose of radiation < 50 Gy or unavailable PD-L1 TPS were excluded. Data on demographics, diagnosis, CRT treatment, durvalumab treatment when applicable, progression and survival were collected. Patients were divided into two groups according to treatments received: CRT + durvalumab and CRT alone. The decision to proceed with adjuvant durvalumab treatment was at the patient’s and treating physician’s discretion, and consent for the treatment plan and schedule was obtained per institutional practice.

The primary outcome was OS, defined as the time between the first radiation treatment and death. The secondary outcome was real-world progression-free survival (PFS), defined as the time between the first radiation treatment and progression identified on imaging, performed at the discretion of the attending physician, or death. OS and PFS were analyzed in two sets of subgroups according to PD-L1 TPS: (1) ≥ 1% vs. < 1%, and (2) PD-L1 TPS ≥ 50% vs. 1–49% and <1%.

Comparisons were made using Chi-square tests for categorical variables and independent t-tests for continuous variables. Kaplan–Meier curves and log-rank tests were used to analyze OS. A multivariable survival model was built with the demographic, diagnostic and treatment-related variables that were either significantly associated with survival in univariate analyses or clinically relevant. A multivariate analysis for survival was also conducted in the CRT + durvalumab group alone to further verify whether PD-L1 was predictive of durvalumab response. Cox proportional hazards model was used for the univariable and multivariable analyses to obtain hazard ratios. Proportionality was assumed as the different covariables do not vary over time and their relationship with survival was assumed to be constant. The data cutoff date was 16 September 2022. For all the analyses, the statistical significance threshold was *p* < 0.05.

This study received approval from the local institutional research ethics board (University of British Columbia—BC Cancer Research Ethics Board; H19-02361), and approval was given for a waiver of consent to extract and analyze the archival data from the database.

## 3. Results

**Figure 1:**
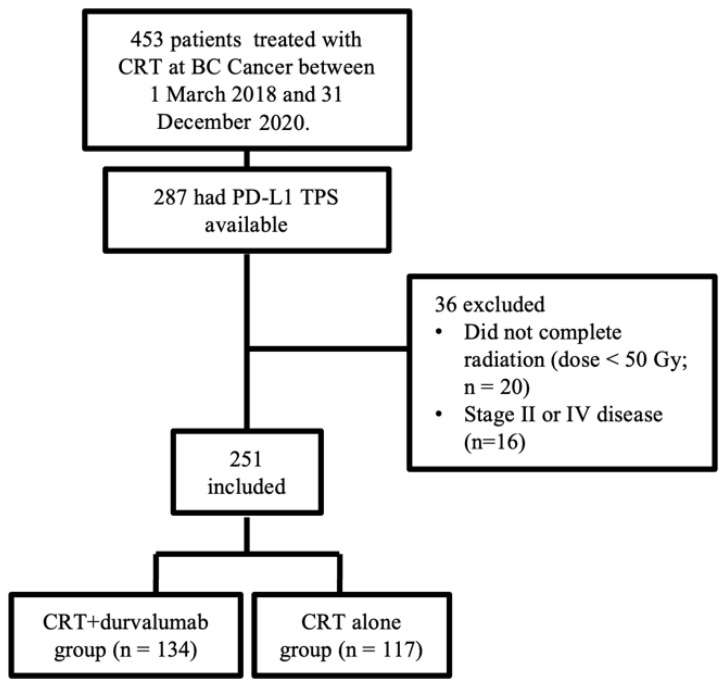
Patient selection flowchart.

Between 1 March 2018 and 31 December 2020, 453 patients with NSCLC were treated with chemoradiotherapy at BC Cancer. Of those, 287 (63%) had available PD-L1 TPS. Thirty-six patients were excluded for either inappropriate stage or incomplete radiation treatment, resulting in a study population of 251 patients, 134 in the CRT + durvalumab group and 117 in the CRT alone group (Figure 1). Patients’ characteristics were mostly well-balanced between groups (Table 1). A statistically significant difference was seen between groups for the smoking history as a result of more active smokers in the CRT + durvalumab group compared to the CRT alone group, which had more previous smokers. Smoking exposure, current or previous, was similar between groups (88.8% vs. 84.6% of patients). PD-L1 TPS was <1%, 1–49% and ≥50% in 76 (30.3%), 70 (27.9%) and 105 (41.8%) patients, respectively, and was not significantly different between the CRT + durvalumab and CRT alone groups. EGFR-activating mutations and ALK fusions were found in 10.4% and 1.6% of patients, respectively. The most frequent genetic alteration was a KRAS mutation, identified in 23.9%. The CRT + durvalumab group had more patients who completed ≥ 2 cycles of chemotherapy (93.2%) compared to the CRT alone group (82.1%). Most patients had a radiation dose ≥ 60 Gy (97.0% and 98.3%). Demographic, pathological and treatment-specific characteristics were overall similar between the PD-L1 ≥ 1% and <1% subgroups, and between treatment arms within those subgroups (Table S1).

The median time between radiation completion and durvalumab start was 40 days. The median treatment duration was 8.0 months (Table 2). No patients were on durvalumab at the data cutoff; 57 patients (42.5%) had completed the year of treatment. The most common reason for stopping was toxicity. When comparing the PD-L1 < 1% and ≥1% subgroups, time to durvalumab initiation was shorter, treatment was longer, and the cumulative dose, as well as the treatment completion rate, were higher in the PD-L1 ≥ 1% subgroup, but those differences were not statistically significant (Table S2).

**Figure 2:**
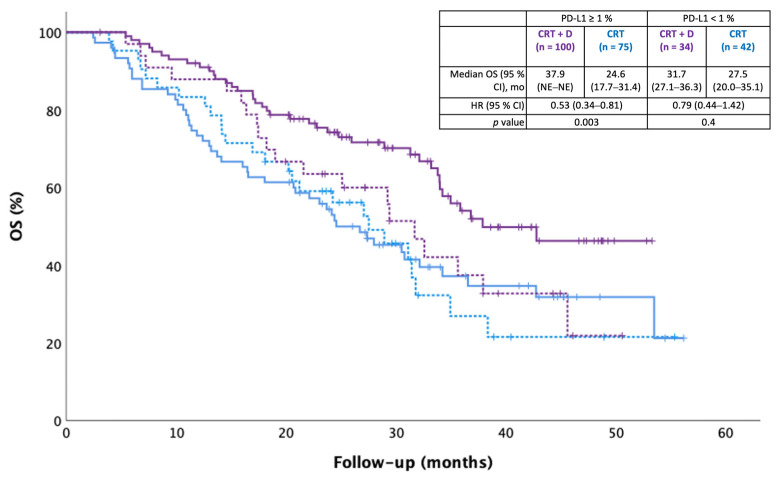
Overall survival (OS) according to PD-L1 expression. OS was defined as the time between the first radiation treatment and death. Date of data cutoff was 16 September 2022. Median follow-up was 27.3 and 23.4 months, respectively.

At the data cutoff on 16 September 2022, after a median follow-up of 27.3 months (CRT + durvalumab) and 23.9 months (CRT alone), 133 patients (47.0%) had died. Median OS was 37.9 months in the CRT + durvalumab group versus 27.4 months in the CRT alone group [HR 0.59 (95% CI 0.42–0.83), *p* = 0.003] (Figure 2). Two-year survival rates were 71.5% versus 56.1%.

Durvalumab was associated with improved OS in the PD-L1 ≥ 1% subgroup [HR 0.53 (95% CI 0.34–0.81), *p* = 0.003], but not in the PD-L1 < 1% subgroup [HR 0.79 (95% CI 0.44–1.42), *p* = 0.4]. Within the PD-L1 ≥ 1% subgroup, the increase in OS was statistically significant in the PD-L1 ≥ 50% of patients, but there was only a positive trend in the PD-L1 1–49% of patients (Figure S1).

**Figure 3:**
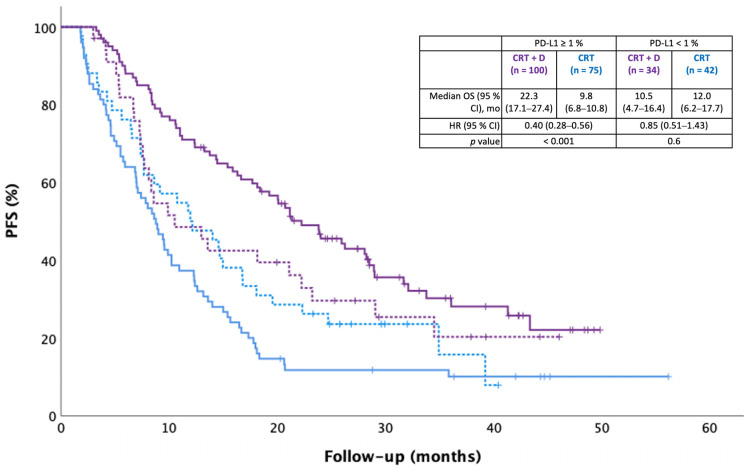
Real-world progression-free survival (PFS) according to PD-L1 expression. PFS was defined as the time between the first radiation treatment and progression or death. Date of data cutoff was 16 September 2022. Median follow-up was 27.3 and 23.4 months, respectively.

Median real-world PFS was 20.8 months in the CRT + durvalumab group and 9.4 months in the CRT alone group [HR 0.51 (95% CI 0.28–0.68), *p* < 0.001]. Durvalumab was associated with significantly longer real-world PFS in the PD-L1 ≥ 1% subgroup (*p* < 0.001), but not in the PD-L1 < 1% subgroup (*p* = 0.6; Figure 3).

We performed univariate analyses with demographic, diagnostic and treatment-related variables from Table 1 and included clinically relevant and/or statistically significant variables in a multivariable survival model (Table 3). There was no multicollinearity, as shown by variance inflation factors between 1.0 and 1.2 for all the included variables. Positive smoking history (*p* = 0.03), including both current and previous smoking, as well as squamous histology (*p* = 0.006), were associated with worse survival in multivariate analyses. When adjusted for age, sex, smoking exposure, histology, stage, PD-L1 TPS, number of chemotherapy cycles and type of platinum, the durvalumab HR for death was 0.56 [(95% CI 0.39–0.80), *p* = 0.002].

Multivariate analysis was conducted in the CRT + durvalumab group only to determine whether PD-L1 TPS was a predictor of OS benefit from durvalumab treatment. Included variables were age, histology, stage and PD-L1 TPS. Compared to PD-L1 < 1%, PD-L1 ≥ 1% had a positive association with survival [HR 0.56 (95% CI 0.32–0.98), *p* = 0.04].

## 4. Discussion

Our retrospective analysis of 251 unresectable stage III NSCLC patients demonstrated longer OS and PFS with CRT + durvalumab compared to CRT alone in PD-L1 ≥ 1%, but not <1% patients. Based on our findings, PD-L1 expression seems to have a predictive value for response to durvalumab, but not a prognostic one.

In the whole cohort, the rate of chemotherapy completion (≥2 cycles) was significantly lower in the CRT arm compared to the CRT + durvalumab arm, putting the CRT arm at a disadvantage. This imbalance was seen in both PD-L1 ≥ 1% and <1% subgroups, although non-significant in the latter. This may be a reflection of frailer patients being less likely to complete chemoradiotherapy and, thus, to obtain durvalumab. Indeed, receiving ≥ 2 cycles of chemotherapy was an inclusion criterion for PACIFIC and, in a sense, selects for more robust patients. The ECOG performance status was not collected, however, which is a limitation of this hypothesis. Another interesting observation is that the PD-L1 ≥ 1% had potentially better durvalumab treatment in terms of time to initiation, treatment duration, cumulative dose and rate of completion when compared to the PD-L1 < 1% subgroup. Although this did not reach statistical significance, it could have contributed to the improved outcomes of the PD-L1 ≥ 1% subgroup on durvalumab.

Our results align with those of the post hoc analysis of PACIFIC, which showed OS benefits from durvalumab in the PD-L1 ≥ 1% [HR 0.59 (95% CI 0.41–0.83)] but not in the PD-L1 < 1% subgroup [HR 1.14 (95% CI 0.71–1.84)] [9]. The magnitude of improvement in OS (HR 0.59) in the PACIFIC study is similar to the one we observed (HR 0.53). Of note is that overall survival was measured from the time of randomization (≤6 weeks of completing CRT) in PACIFIC, whereas we measured it from the first radiation treatment. The predictive value of PD-L1 expression for durvalumab response is concordant with the results of a recently published retrospective analysis of 312 patients treated with adjuvant durvalumab after CRT in 2017–2021 [13]. Improved OS and PFS were seen with every absolute increase of 25% in PD-L1 TPS, and in the PD-L1 ≥ 50% and 1–49% subgroups compared to the <1% subgroup. Furthermore, in PACIFIC-R, a retrospective study of 1399 patients started on durvalumab in 2017–2018, real-world PFS was longer in PD-L1 ≥ 1% (22.4 months) compared to <1% (15.6 months) patients. [14]. Two smaller retrospective analyses of patients who received durvalumab between 2017 and 2020 demonstrated that, compared to PD-L1 < 1%, PD-L1 ≥ 50% patients had improved outcomes [15,16]. In both studies, however, the difference between the 1–49% and <1% groups was not significant. Finally, based on the multivariate survival analysis in the whole cohort, our study did not demonstrate a prognostic role of PD-L1, which confirms prior data in locally advanced NSCLC treated with CRT [17,18,19].

Our results should be interpreted in light of our study’s limitations. Variables not accounted for by chart review could have caused unmeasured imbalances between groups and biased the survival analyses. Real-world PFS is an imperfect outcome because of the lack of standardized, timed imaging. Progression was likely underestimated and detected late compared to a clinical trial setting, but this would have affected the whole cohort and is unlikely to have biased comparisons between groups.

## 5. Conclusions

In conclusion, our study demonstrated improved OS and PFS with durvalumab in PD-L1 ≥ 1%, but not in PD-L1 < 1% stage III NSCLC after CRT. Notwithstanding limitations inherent to the retrospective design and the need for a prospective study, those results align with previously published data in the field. PD-L1 TPS should be weighed in the balance when it comes to prescribing adjuvant durvalumab, along with the patient’s unique set of comorbidities, performance status and goals of care.

## Figures and Tables

**Table 1:** Patients characteristics.

	CRT + Durvalumab (*n* = 134)	CRT Alone (*n* = 117)	*p* Value
Age, years	66 ± 8	68 ± 8	0.07
Sex			0.06
Male	80 (59.7%)	56 (47.9%)	
Female	54 (40.3%)	61 (52.1%)	
Ethnicity			0.2
Asian	11 (8.2%)	16 (13.7%)	
Non-Asian	123 (91.8%)	101 (86.3%)	
Smoking history			0.02
Current	41 (30.6%)	18 (15.4%)	
Past	78 (58.2%)	81 (69.2%)	
Never	15 (11.2%)	18 (15.4%)	
Living area			0.8
Urban	111 (83.5%)	98 (84.5%)	
Rural	22 (16.5%)	18 (15.5%)	
Histology			0.6
Squamous	39 (28.3%)	26 (22.2%)	
Non-squamous	94 (70.1%)	88 (75.2%)	
Other	3 (2.2%)	3 (2.6%)	
Stage			0.8
IIIA	72 (53.7%)	63 (53.8%)	
IIIB	54 (40.3%)	49 (41.9%)	
IIIC	8 (6.0%)	5 (4.3%)	
EGFR-activating mutation			0.7
Positive	13 (9.7%)	13 (11.1%)	
Negative	84 (62.7%)	77 (65.8%)	
Unknown	37 (27.6%)	27 (23.1%)	
Other driver mutations			0.7
ALK	2 (1.5%)	2 (1.7%)	
ROS-1	0	0	
KRAS	32 (23.9%)	28 (23.9%)	
HER2	4 (3.0%)	3 (2.6%)	
BRAF	1 (0.7%)	4 (3.4%)	
cMET exon 14 skip	2 (1.5%)	1 (0.9%)	
Other	0	2 (1.7%)	
None	27 (20.1%)	18 (15.4%)	
Unknown	53 (39.6%)	46 (39.3%)	
PD-L1 TPS			0.2
<1%	34 (25.4%)	42 (35.9%)	
1–49%	40 (29.9%)	30 (25.6%)	
≥50%	60 (44.8%)	45 (38.5%)	
Platinum type			0.5
Cisplatin	50 (37.3%)	39 (33.3%)	
Carboplatin	84 (62.7%)	78 (66.7%)	
≥2 cycles *	124 (93.2%)	96 (82.1%)	0.007
Radiation			
Dose, Gy	60 ± 2	60 ± 1	0.6
Dose ≥ 60 Gy	130 (97.0%)	115 (98.3%)	0.5

Data are presented as mean ± SD, median (range) and *n* (%). * For the purpose of this analysis, when weekly carboplatin–paclitaxel was used, three weekly treatments were counted as one cycle. PD-L1 = programmed death-ligand 1, TPS = tumor proportion score, Gy = grays.

**Table 2:** Durvalumab treatment characteristics (*n* = 134).

Radiation completion to durvalumab start, days	40 (13–186)
≤42 days	72 (53.7%)
Treatment duration, months	8.0 (0.0–15.0)
Cumulative dose, mg/kg	165 (10–270)
Treatment status	
Ongoing	0
Completed	57 (42.5%)
Stopped	
Progression	29 (21.6%)
Toxicity	35 (26.1%)
Other reason	11 (8.2%)
Unknown	2 (1.5%)

Data are expressed as *n* (%) or median (range).

**Table 3:** Multivariate survival model (*n* = 251).

	Univariate Analyses HR (95% CI)	*p* Value	Multivariate Analyses HR (95% CI)	*p* Value
Age	1.01 (1.00–1.04)	0.07	1.01 (0.98–1.03)	0.6
Male sex	1.45 (1.03–2.05)	0.04	1.31 (0.89–1.91)	0.2
Positive smoking history	2.11 (1.14–3.91)	0.02	2.03 (1.07–3.86)	0.03
Squamous histology (vs. non-squamous)	1.99 (1.39–2.86)	<0.001	1.75 (1.17–2.60)	0.006
Stage (vs. IIIA)		0.2		0.3
IIIB	1.30 (0.92–1.85)	0.1	1.35 (0.94–1.94)	0.1
IIIC	1.53 (0.74–3.20)	0.3	1.25 (0.58–2.70)	0.6
PD-L1 TPS > 1% (vs. < 1%)	0.74 (0.51–1.05)	0.09	0.80 (0.55–1.16)	0.2
Cisplatin-based chemotherapy	0.77 (0.54–1.1)	0.2	0.92 (0.62–1.37)	0.7
≥2 cycles of chemotherapy	0.67 (0.42–1.08)	0.1	0.87 (0.52–1.46)	0.6
Durvalumab	0.59 (0.42–0.83)	0.003	0.56 (0.39–0.80)	0.002

PD-L1 = programmed death-ligand 1, TPS = tumor proportion score.

## Data Availability

The data presented in this study are available on request from the corresponding author.

## Supplementary Materials

The following supporting information can be downloaded at: https://www.mdpi.com/article/10.3390/curroncol30080543/s1, Table S1 Patients characteristics in PD-L1 ≥ 1% and <1% subgroups, Table S2 Durvalumab treatment characteristics in PD-L1 ≥ 1% and <1% subgroups, Figure S1. Overall survival (OS) according to PD-L1 expression ≥ 50%, 1–49% and <1%.
