# Supplementary material for: Beyond PACIFIC: Real-World Outcomes of Adjuvant Durvalumab According to Treatment Received and PD-L1 Expression
Source: Curr Oncol. 2023 Aug 8;30(8):7499–507. doi: 10.3390/curroncol30080543 (PMC10453050; doi:10.3390/curroncol30080543)
Supplement: Supplementary file 1 [file curroncol-30-00543-s001.zip › curroncol-2461494-supplementary.pdf]

Supplementary materials

Table S1 Patients characteristics in PD-L1 ≥ 1% and < 1% subgroups

	PD-L1 < 1 %			PD-L1 ≥ 1 %		
	**CRT+D (n = 34)**	**CRT (n = 42)**	**p value**	**CRT+D (n = 100)**	**CRT (n = 75)**	**p value**
**Age, years**	66 ± 8	67 ± 9	0.6	66 ± 8	69 ± 8	0.05
**Sex**			1.0			0.01
**Male**	21 (61.8 %)	26 (61.9 %)		59 (59.0 %)	30 (40.0 %)	
**Female**	13 (38.2 %)	16 (38.1 %)		41 (41.0 %)	45 (60.0 %)	
**Ethnicity**			0.5			0.03
**Asian**	4 (11.8 %)	3 (7.1 %)		7 (7.0 %)	13 (17.3 %)	
**Non-Asian**	30 (88.2 %)	39 (92.9 %)		93 (93.0 %)	62 (82.7 %)	
**Smoking history**			0.2			0.04
**Current**	14 (41.2 %)	9 (21.4 %)		27 (27.0 %)	9 (12.0 %)	
**Past**	16 (47.1%)	28 (66.7 %)		62 (62.0 %)	53 (70.7 %)	
**Never**	4 (11.8 %)	5 (11.9 %)		11 (11.0 %)	13 (17.3 %)	
**Living area**			0.4			0.3
**Urban**	29 (85.3 %)	32 (78.0 %)		82 (82.8 %)	66 (88.0 %)	
**Rural**	5 (14.7 %)	9 (22.0 %)		17 (17.2 %)	9 (12.0 %)	
**Histology**			0.8			0.6
**SCC**	11 (32.4 %)	11 (26.2 %)		26 (26.0 %)	15 (20.0 %)	
**Non-SCC**	22 (64.7 %)	30 (71.4 %)		72 (72.0 %)	58 (77.3 %)	
**Other**	1 (2.9 %)	1 (2.4 %)		2 (2.0 %)	2 (2.7 %)	
**Stage**			0.3			0.3
**IIIA**	20 (58.8 %)	28 (66.7 %)		52 (52.0 %)	35 (46.7 %)	
**IIIB**	14 (41.2 %)	12 (28.6 %)		40 (40.0 %)	37 (49.3 %)	
**IIIC**	0	2 (4.8 %)		8 (8.0 %)	3 (4.0 %)	
**EGFR mutation**			0.7			0.4
**Positive**	5 (14.7 %)	4 (9.5 %)		8 (8.0 %)	9 (12.0 %)	
**Negative**	17 (50.0 %)	24 (57.1 %)		67 (67.0 %)	53 (70.7 %)	
**Unknown**	12 (35.3 %)	14 (33.3 %)		25 (25.0 %)	13 (17.3 %)	
**Other driver**			0.6			0.8
**mutations**	1 (2.9 %)	0		1 (1.0 %)	2 (2.7 %)	
**ALK**	0	0		0	0	
**ROS-1**	6 (17.6 %)	7 (16.7 %)		26 (26.0 %)	21 (28.0 %)	
**KRAS**	2 (5.9 %)	1 (2.4 %)		2 (2.0 %)	2 (2.7 %)	
**HER2**	0	2 (4.8 %)		1 (1.0 %)	2 (2.7 %)	
**BRAF**	0	0		2 (2.0 %)	1 (1.3 %)	
**cMET exon 14 skip**	0	2 (4.8 %)		0	0	
**Other**	6 (17.6 %)	8 (19.0 %)		21 (21.0 %)	10 (13.3 %)	
**None**	14 (41.2 %)	18 (42.9 %)		39 (39.0 %)	28 (37.3 %)	
**Unknown**						

**Platinum**			0.8			0.3
**Cisplatin**	11 (32.4 %)	15 (35.7 %)		39 (39.0 %)	24 (32.0 %)	
**Carboplatin**	23 (67.6 %)	27 (64.3 %)		61 (61.0 %)	51 (68.0 %)	
**≥ 2 cycles**	31 (91.2 %)	33 (78.6 %)	0.1	93 (93.9 %)	63 (84.0 %)	0.03
**Radiation**						
**Dose, Gy**	60 ± 1	60 ± 1	0.07	60 ± 2	60 ± 1	0.9
**Dose ≥ 60 Gy**	32 (94.1 %)	42 (100 %)	0.1	98 (98.0 %)	73 (97.3 %)	0.8

Data are presented as mean ± SD and n (%). CRT = chemoradiotherapy, CRT+D = chemoradiotherapy+durvalumab, ECOG = Eastern Cooperative Oncology Group, PS = performance status, PD-L1 = programmed death-ligand 1, TPS = tumor proportion score, Gy = grays.

Table S2 Durvalumab treatment characteristics in PD-L1 ≥ 1% and < 1% subgroups

	**PD-L1 < 1 % (n = 34)**	**PD-L1 ≥ 1 % (n = 100)**	**p value**
**Radiation completion to durvalumab start, days**	46 (17-91)	40 (13-186)	0.3
**≤ 42 days**	16 (47.1 %)	56 (56.0 %)	0.4
**Treatment duration, months**	6.4 (0.0-14.4)	8.9 (0.0-15.0)	0.1
**Cumulative dose, mg/kg**	145 (10-260)	175 (10-270)	0.2
**Treatment status**			0.8
**Ongoing**	0	0	
**Completed**	12 (35.3 %)	45 (45.0 %)	
**Stopped**			
**Progression**	9 (26.5 %)	20 (20.0 %)	
**Toxicity**	9 (26.5 %)	26 (26.0 %)	
**Other reason**	3 (8.8 %)	8 (8.0 %)	
**Unknown**	1 (2.9 %)	1 (1.0 %)	

Data are expressed as n (%) or median (range).

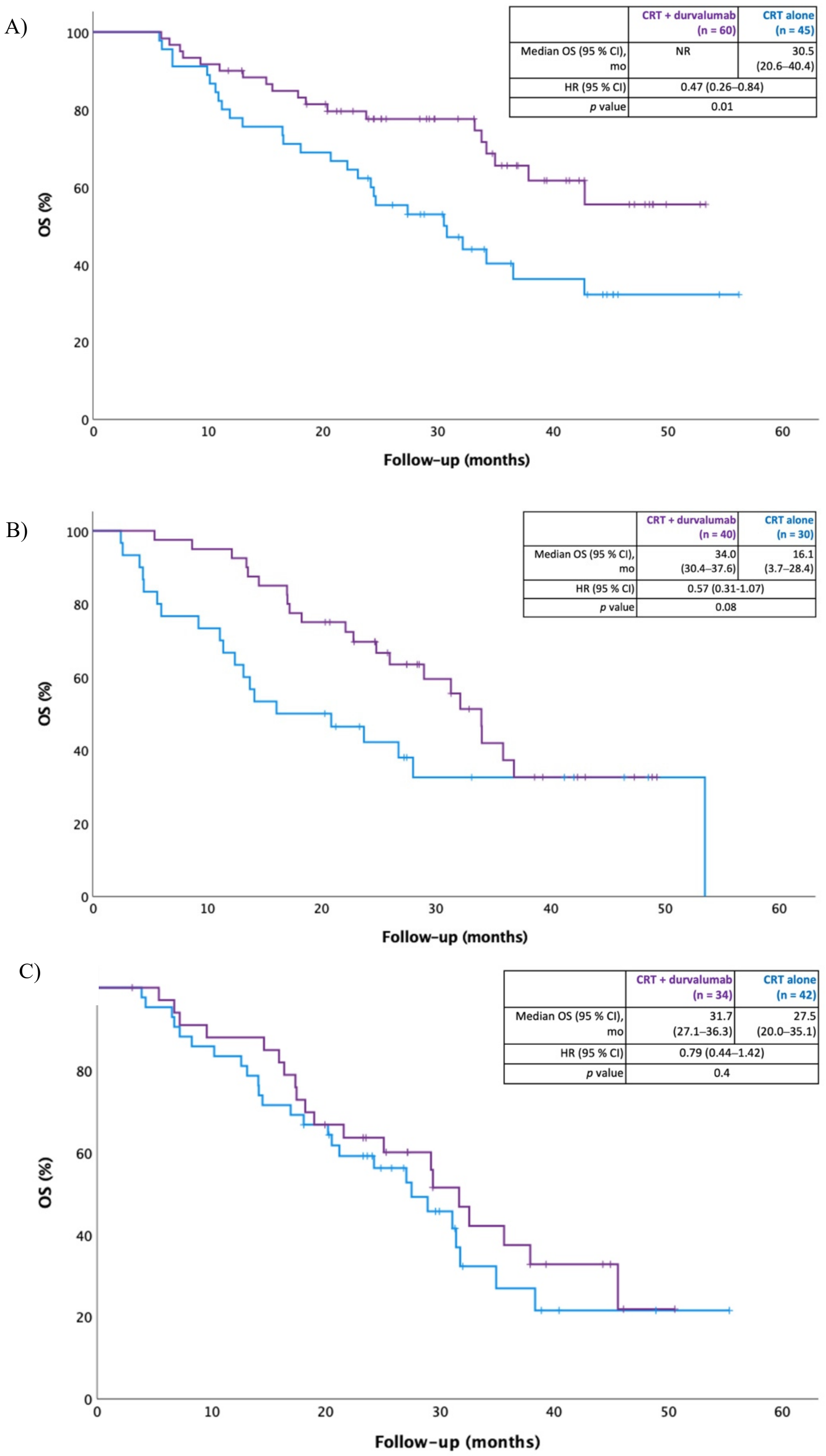

Figure S1. Overall survival (OS) according to PD-L1 expression ≥ 50 %, 1-49 % and < 1%

OS was defined as the time between the first radiation treatment and death. Date of data cutoff was September 16, 2022. Median follow-up was 27.3 and 23.9 months, respectively. A) PD-L1 TPS ≥ 50 % (n = 105), B) PD-L1 TPS 1-49 % (n = 70) and C) PD-L1 TPS < 1 % (n = 76).
